# Metabolic Effects of Loquat Juice (*Eriobotrya japonica* Lindl *Mkarkeb* Variety) on Lipid Homeostasis, Liver Steatosis, and Oxidative Stress in Hyperlipidemic Mice Fed a High-Fat–High-Fructose Diet

**DOI:** 10.3390/metabo14110592

**Published:** 2024-11-02

**Authors:** Imane Mokhtari, Thamer Aljutaily, Huda Aljumayi, Khadija S. Radhi, Abdulkarim S. Almutairi, Hassan Barakat, Ibrahim Khalifa, Souliman Amrani, Hicham Harnafi

**Affiliations:** 1 Laboratory of Bioresources, Biotechnologies, Ethnopharmacology and Health, Faculty of Sciences, University Mohamed I, Oujda 60 000, Morocco; mokhtari.imane@ump.ac.ma (I.M.); s.amrani@ump.ac.ma (S.A.); h.harnafi@ump.ac.ma (H.H.); 2 Department of Food Science and Human Nutrition, College of Agriculture and Food, Qassim University, Buraydah 51452, Saudi Arabia; thamer.aljutaily@qu.edu.sa; 3 Department of Food Science and Nutrition, College of Sciences, Taif University, P.O. Box 11099, Taif 21944, Saudi Arabia; huda.a@tu.edu.sa (H.A.); ksradhi@tu.edu.sa (K.S.R.); 4 Al Rass General Hospital, Qassim Health Cluster, Ministry of Health, King Khalid District, Al Rass, Saudi Arabia; abdulkarimsa@moh.gov.sa; 5 Food Technology Department, Faculty of Agriculture, Benha University, Moshtohor 13736, Egypt; ibrahiem.khalifa@fagr.bu.edu.eg

**Keywords:** lipid metabolism, loquat juice, hepatic steatosis, high-fat/high fructose diet, fruit, food supply

## Abstract

Background: Loquat fruit is consumed for its flavorful taste and a rich array of health-promoting compounds like phenolics, flavonoids, and carotenoids. This study aimed at the biochemical characterization of fresh juice from the Moroccan *Mkarkeb* variety of loquat and evaluating its effects on lipid homeostasis and liver steatosis in hyperlipidemic mice. Methods: The biochemical characterization followed AOAC methods. In vivo study involved hyperlipidemic mice fed a high-fat, high-fructose diet for 6 weeks and treated with loquat juice at 3.5 and 7 mL kg^−1^ or fenofibrate at 4 mg·kg^−1^. The concentrations of lipids in plasma, liver, adipose tissue, feces, and bile and blood glucose levels were quantified. Liver steatosis was visually examined and confirmed histologically, and liver injury markers (AST, ALT, ALP, LDH, and TB) were measured. Liver oxidative stress was assessed by measuring MDA content and antioxidative enzyme activities. Results: Our findings indicate that fresh loquat juice is poor in fat and protein and contains moderate sugars with a low energy value (40.82 ± 0.25 kcal/100 g). It is also rich in minerals, vitamin C, phenolic acids, flavonoids, and carotenoids. The juice effectively restored lipid metabolism by enhancing reverse cholesterol transport and lowering LDL-cholesterol, triglycerides, and the atherogenic index. The studied juice decreases blood glucose and prevents weight gain and lipid accumulation in the liver and adipose tissue. The juice prevents lipotoxicity-induced liver injury, corrects toxicity markers, and improves the liver’s morphological and histological structures. It also reduces oxidative stress by lowering MDA and activating SOD and catalase. Conclusions: The juice holds high nutritional and medicinal value, potentially preventing lipid disorders and cardiovascular issues.

## 1. Introduction

Loquat (*Eriobotrya japonica* Lindl), a member of the Rosaceae family, is an evergreen fruit tree that originated in China. It is now widely cultivated in temperate and subtropical regions worldwide, including the Mediterranean, Turkey, Pakistan, India, and Brazil [1]. In Morocco, loquat cultivation spans approximately 600 hectares in the Berkane province of Oriental Morocco, accounting for more than 80% of the country’s loquat-growing area. This cultivation is concentrated in the communes of Zegzel, Takerboust, Tazaghine, and Ouaoullout, where four loquat varieties are produced: Tanaka, Navela, Muscat, and *Mkarkeb*. The Japanese variety *Mkarkeb* is known for its large size, firm yellow-orange flesh, sweet and juicy flavor, few small seeds, excellent taste quality, and late maturity. Since 2013, Zegzel loquat has been recognized with the “Protected Geographical Indication” (PGI) status. It is a vital focus of the regional agricultural development plan to promote local products from the Oriental region [2].

Loquat fruit is esteemed for its health benefits, taste, and high phytochemical content, including polyphenols and carotenoids [3]. While it is usually enjoyed fresh, the mature loquat fruit has recently found favor as a critical ingredient in jams and jellies, owing to its nutritional richness and appealing flavor profile [4]. Like many other fruits, the taste quality of loquat is closely linked to its sugar content and the ratio of total soluble solids to titratable acidity (TSS/TA ratio) [5]. Nutritionally, loquat is rich in sugars such as glucose, fructose, and sucrose, and it also contains dietary fibers, organic acids, vitamins, and minerals [3]. Loquat is also a significant source of various nutritional bioactive compounds, including polyphenols, carotenoids, and triterpenoids, known for their pharmacological activities [3]. Numerous experimental studies have demonstrated the potential health benefits of loquat in animal and cell line models, such as its hypoglycemic, anti-inflammatory, antioxidative, and hypolipidemic properties [4]. However, further research is needed to better understand the correlation between these bioactive compounds and their biological activities.

Cardiovascular diseases (CVD) impact over 100 million people globally [6]. The primary cause of CVD is atherosclerosis, a complex and multifactorial condition characterized by hyperlipidemia, oxidative stress, and inflammation [7]. It is well established that dyslipidemia, particularly elevated levels of plasma low-density lipoproteins (LDL) and low levels of high-density lipoproteins (HDL), is a major risk factor for atherosclerosis [8]. Oxidative stress leads to the oxidation of LDL, marking the initial stage of the atherogenic process [9]. Additionally, oxidative stress associated with hypercholesterolemia increases the production of free radicals, which contribute to atherosclerosis and cause biological alterations such as lipid peroxidation, protein oxidation, and reduced activity of antioxidant defenses [10]. Moreover, hyperlipidemia can result in excessive lipid accumulation in various tissues, including the liver and kidneys, which leads to cellular lipotoxicity [11]. To study hyperlipidemia and related metabolic disorders, researchers commonly use animal models induced by high-fat and high-fructose diets [12]. Conversely, numerous epidemiological and preclinical studies have underscored the benefits of the Mediterranean diet. This diet, rich in fruits, vegetables, whole grains, healthy fats, and lean proteins, has been linked to a lower incidence of obesity, liver disorders, and metabolic syndrome in human populations [13].

Despite previous studies on loquat’s biochemical composition, little attention has been given to the unique characteristics of the *Mkarkeb* variety. This study aims to fill that gap by identifying and quantifying the specific bioactive compounds present in *Mkarkeb* loquat, potentially distinguishing it from other varieties. Notably, our investigation may reveal unique compounds that enhance its health benefits, particularly regarding lipid metabolism and cardiovascular protection. Understanding these differences can provide insights into the fruit’s applications in nutrition and health, contributing valuable knowledge to the field.

In this context, the present study analyzes the biochemical composition of loquat fruit (variety *Mkarkeb*) cultivated in Eastern Morocco. Notably, to the best of our knowledge, this is the first study to explore this specific variety of loquat fruit in such detail.

## 2. Materials and Methods

### 2.1. Plant Material

Ripe loquat fruits (*Eriobotrya japonica* (Thunb.) Lindl., Variety *Mkarkeb*) were collected from the Zegzel Valley in Berkane Province, Oriental Morocco, in May 2023. A taxonomist identified the fruits, and a voucher specimen was deposited under the collection number E.J.10.

### 2.2. Morphologic Characteristics and Juice Preparation

Loquat fruits were chosen based on their intact appearance, consistent size, and uniform color. To assess their morphological traits—such as average fresh weight, height (from the tallest point), width (at the broadest point), and thickness—ten fruits were grouped, and the averages for each parameter were calculated. A digital balance was used to measure the weight of each fruit, while Vernier calipers were employed to determine their length and diameter. The shape index, which is the length-to-width ratio, was calculated by dividing the fruit’s length by its diameter. Loquat juice preparation followed the method outlined by Meng et al. [14] with minor modifications; fruits were thoroughly rinsed with tap water, peeled, and deseeded. The loquat flesh was pulped using an electric blender and filtered, and fresh juice was obtained for biochemical analyses, while the remaining juice was stored at −20 °C. Approximately 400 g of juice and 600 g of residue were obtained from each kilogram of loquat flesh.

### 2.3. Determination of the Nutritional Composition of Loquat Juice

#### 2.3.1. Analysis of Physicochemical Properties of Fresh Loquat Juice

The pH of the fresh juice was measured using a pH meter. Total soluble solids (TSS) were determined with a refractometer and reported in °Brix (AOAC 920.151). Titrable acidity (TA) was assessed by titrating the fresh juice with a 0.1 N sodium hydroxide solution, and the results were expressed as grams of malic acid equivalent per 100 g of juice, using phenolphthalein (1%) as an indicator (AOAC 943.03). The maturity index, calculated as the TSS/TA ratio, was also determined [5]. All measurements were performed in triplicate.

#### 2.3.2. Measurement of Total Sugar Content and Sweetness Index in Loquat Juice

Total sugar was quantified using the phenol-sulfuric acid method [15]. Fresh juice was diluted, combined with phenol (5%) and sulfuric acid, and the absorbance was read at 490 nm. The total sugar content was calculated from a standard curve made with D-fructose, expressed in grams per 100 g of juice. Sugars in the loquat juice were analyzed by HPLC as described by Yu et al. [16]. The sweetness index was estimated based on the relative sweetness of fructose, glucose, and sucrose [17].

#### 2.3.3. Analysis of Organic Acids in Loquat Juice

Organic acids in loquat juice were analyzed following a modified protocol from Deng et al. [18]. Diluted juice was filtered, and 10 μL was injected into a C18 column under specific chromatographic conditions. Quantification was done using standard calibration curves, and the results were reported in milligrams per 100 g of juice.

#### 2.3.4. Determination of Fat and Protein Content in Loquat Juice

Fat content was measured using the Soxhlet extraction method (AOAC 963.15), while protein content was determined following the AOAC 920.152 procedure.

#### 2.3.5. Ash and Mineral Contents

The ash content was determined using a gravimetric method (AOAC 940.26). Precisely, 25 g of loquat juice was placed into a pre-weighed porcelain crucible and then heated in a muffle furnace at 525 °C for 2 h. Afterward, the crucible was removed, cooled in a desiccator, and reweighed to calculate the ash content, expressed as grams per 100 g of juice. The mineral composition of the ash sample was analyzed via atomic spectrometry (AOAC 968.08).

#### 2.3.6. Crude Fiber Contents

The crude fiber content was determined following the Weende method (AOAC 978.10). To begin, 5 g of previously dried juice were treated with a 1.25% sulfuric acid solution to isolate the fibers, after which the mixture was filtered and rinsed. The fibers were subsequently treated with a 1.25% sodium hydroxide solution, filtered, and rinsed again. The fibers were then dried in an oven at 105 °C for one hour. This final residue, containing both crude fibers and ash, was weighed, with the ash content subtracted to isolate the fiber content. Results are presented as grams of fiber per 100 g of juice.

#### 2.3.7. Total Polyphenols, Flavonoids, Carotenoids and Vitamin C

The total polyphenol content in loquat juice was estimated using the Folin–Ciocalteu method, as described by Mokhtari et al. [19]. A 0.5 mL sample of diluted loquat juice was combined with 0.25 mL of Folin–Ciocalteu reagent and 0.5 mL of 20% aqueous sodium carbonate solution. After incubating the mixture for 30 min at room temperature in the dark, the absorbance was recorded at 725 nm. Polyphenol levels were quantified using a calibration curve created with chlorogenic acid, with results expressed in milligrams per gram of juice.

Flavonoid content was measured using a previously established method [19]. The concentration was determined by comparing the results with a calibration curve from rutin standard solutions, and values are presented in milligrams per gram of juice.

Individual phenolic compounds in the juice were analyzed by HPLC according to a prior method [20]. In brief, 10 µL of appropriately diluted juice was injected into a C18 column (250 mm × 4.6 mm, 5 µm particle size). Elution was performed with a gradient of ultrapure water with 0.5% acetic acid (A) and acetonitrile (B) at a flow rate of 1 mL per minute and a column temperature of 20 °C. The gradient steps were as follows: 0–15 min: 80% A, 20% B; 15–20 min: 60% A, 40% B; 20–35 min: 40% A, 60% B; and 35–40 min: 95% A, 5% B. Chromatograms were observed at 340 nm, and compound identification was based on retention times and UV-visible spectra, using a standard phenolic compound database as a reference.

Carotenoid content was determined following a slightly modified method from Kabiri et al. [2]. Fifty milliliters of loquat juice was extracted three times with 50 mL of n-hexane in a separating funnel. After allowing the two layers to separate for 30 min, the absorbance of the carotenoid-containing organic phase was measured at 470 nm, and carotenoid content was calculated using a β-carotene standard curve.

Vitamin C was quantified using a 2,6-dichlorophenol-indophenol (DPIP) titration method, as described by Kabiri et al. [2] with modifications. Ten milliliters of diluted juice was mixed with 1 mL of glacial acetic acid and titrated to a light, stable pink color. The vitamin C amount was calculated using a standard curve prepared with L-ascorbic acid.

#### 2.3.8. Energy Value

The total energy content of loquat fruit was calculated using standard Atwater factors, which assign 4 kcal per gram of protein, 9 kcal per gram of fat, and 4 kcal per gram of carbohydrates. The caloric value was determined by summing the products of the respective nutrient weights:

Energy value (kcal 100 g^−1^) = (Protein (g) × 4) + (Carbohydrate (g) × 4) + (lipid (g) × 9).

### 2.4. Effect of Mkarkeb Loquat Juice on Mice Fed a High-Fat, High-Fructose Diet (HFFD)

#### 2.4.1. Animals and Treatment

Male Swiss *Albino* mice (27–28 g) were bred at the Faculty of Sciences, University Mohammed I in Oujda, Morocco. The mice were housed under standard conditions at 22 °C with a 12 h light/dark cycle and had unrestricted access to food and water. The animal experiments were conducted according to the Care and Use of Laboratory Animals Guidelines set forth by the US National Institutes of Health (NIH Publication No. 85–23, revised 1996) and approved by the Institutional Review Board of the Faculty of Sciences, Oujda, Morocco (approval number: 002023) issued on 10 May 2023.

#### 2.4.2. Animal Diet

The high-fat–high-fructose diet (HFFD) was prepared with minor modifications based on the method described by Yustisia et al. [21]. A standard mice diet from the Society Alf Sahel, Meknes, Morocco, was combined with specific amounts of beef fat (16%), cholesterol (1.5%), fructose (10%), egg yolk (10%), and deoxycholic acid (0.2%).

#### 2.4.3. Experimental Design

After a 2-week acclimatization period, the mice were randomly assigned to one of six groups, each consisting of eight animals, and housed in individual cages. The experimental groups were treated as follows: NC (Normal control): fed a standard diet and daily gavaged with distilled water. JCG (Juice Control): fed a standard diet and daily gavaged with 0.5 mL of loquat fresh juice (7 mL kg^−1^) for 6 weeks. HC (Hyperlipidemic Control): fed a high-fat, high-fructose diet (HFFD) and daily gavaged with distilled water for 6 weeks. JTG (3.5 mL kg^−1^): fed the HFFD and daily gavaged with loquat juice at 3.5 mL kg^−1^. JTG (7 mL kg^−1^): fed the HFFD and daily gavaged with loquat juice at 7 mL kg^−1^. FG (Fenofibrate Group): fed the HFFD and daily gavaged with fenofibrate at 4 mg kg^−1^. Food consumption was recorded daily, and body weight was recorded every fortnight.

#### 2.4.4. Fecal Samples, Blood and Organ Collection

Fecal samples were collected at 2, 4, and 6 weeks and stored at −20 °C. Prior to sampling, all animals were fasted overnight and lightly anesthetized with diethyl ether. Blood samples were obtained using trisodium citrate, then centrifuged at 3000 rpm for 15 min to separate the plasma used for biochemical analyses. After the animals were sacrificed, the liver and abdominal adipose tissues were carefully removed, washed with a cold saline solution, and weighed. The weights were reported as grams per 100 g of body weight.

#### 2.4.5. Plasma Biochemical Analysis

Plasma levels of total cholesterol, LDL-cholesterol, HDL-cholesterol, triglycerides, glucose, aspartate aminotransferase (AST), alanine aminotransferase (ALT), alkaline phosphatase (ALP), lactate dehydrogenase (LDH), and total bilirubin (TB) were assessed using biomedical kit methods. The detailed procedures for these analyses can be found in our previous studies [19,20]. Lipid indices were determined using established formulas, as outlined in prior research by Mokhtari et al. [20].

#### 2.4.6. Analysis of Hepatic, Adipose Tissue, and Fecal Lipids

Lipid extraction from the liver, abdominal adipose tissue, and fecal matter were conducted following the method described by Mokhtari et al. [19].

#### 2.4.7. Biliary Cholesterol Analysis

Bile was obtained from the gall bladder through the use of a 30-gauge needle. The assessment of biliary cholesterol levels used an identical method to analyze total cholesterol in plasma.

#### 2.4.8. Liver Histology

Fresh animal tissues were cut into approximately 1 cm squares, fixed in a buffered formalin solution (10%), dehydrated, and subsequently embedded in paraffin. The tissues were then sliced into 4 to 5 µm thick sections using a microtome. These histological sections underwent cleaning with toluene hydration through decreasing alcohol baths and were ultimately stained with hematoxylin and eosin. The prepared samples were then mounted on glass slides using a light microscope for histological scrutiny.

#### 2.4.9. Oxidative Stress Biomarker: Liver Malondialdehyde Content, SOD, and Catalase Activities

The liver lipid peroxidation was estimated by measuring the content of malondialdehydes (MDA) using our previously published method [20]. Liver SOD and catalase activities were determined following the modified method of Yaribeygi [22].

### 2.5. Statistical Analysis

The obtained data were presented as mean ± standard error of the mean (SEM) and compared by one-way variance analysis (ANOVA). This statistical analysis was executed using GraphPad Prism 9.5.0 software, with significance at *p* < 0.05.

## 3. Results

### 3.1. Nutritional Composition of Loquat Fresh Juice

Table 1 details various attributes of mature loquat juice, including physical dimensions and chemical properties. The average weight of the mature fruit is 68.73 ± 2.19 g, with a length of 41.27 ± 1.30 mm and a width of 56.09 ± 2.44 mm. The fruit thickness measures 24.08 ± 1.94 mm, and the fruit shape index is 0.73 ± 0.13, indicating the fruit’s relative proportions. The juice has a pH of 3.71 ± 0.22 and a titrable acidity of 0.64 ± 0.03%, contributing to its overall tartness. Total soluble solids (TSS) are 12.40 ± 0.25 °Brix, and the TSS/TA ratio is 19.37 ± 0.17, suggesting a well-balanced sweetness-to-acidity ratio. The total sugar content is 9.71 ± 0.07 g 100 g^−1^, with fructose and glucose levels of 4.72 ± 0.28 g/100 g and 2.44 ± 0.34 g 100 g^−1^, respectively. Sucrose was not detected in the juice. The sweetness index is 16.15 ± 0.31, reflecting the perceived sweetness of the juice. Nutritionally, the juice is low in fat (0.052 ± 0.0029 g 100 g^−1^) and protein (0.38 ± 0.04 g 100 g^−1^), with minimal crude fiber (0.11 ± 0.06 g 100 g^−1^). The predominant organic acid is malic acid, present at 537.08 ± 11.04 mg 100 g^−1^, followed by tartaric acid (48.02 ± 3.14 mg 100 g^−1^), succinic acid (16.10 ± 1.84 mg 100 g^−1^), and oxalic acid (9.10 ± 1.06 mg 100 g^−1^). Vitamin C content is 7.10 ± 0.08 mg 100 g^−1^, and carotenoids are 58.01 ± 4.05 µg g^−1^. Mineral content includes potassium (259.01 ± 2.01 mg 100 g^−1^), sodium (59.07 ± 1.40 mg 100 g^−1^), phosphorus (23.10 ± 1.50 mg 100 g^−1^), calcium (19.10 ± 1.01 mg 100 g^−1^), magnesium (18.29 ± 0.99 mg 100 g^−1^), and iron (2.91 ± 0.10 mg 100 g^−1^). The energy value of the juice is 40.82 ± 0.25 kcal 100 g^−1^. The total polyphenol content is 138.08 ± 0.71 mg 100 g^−1^, and flavonoids are at 85.18 ± 4.53 mg 100 g^−1^. The mature *Mkerkeb* loquat juice is characterized by a low fat and protein content, moderate sweetness, and notable levels of organic acids, vitamins, minerals, and bioactive compounds, contributing to its nutritional and health-promoting potential.

### 3.2. HPLC Analysis of Loquat Juice Polyphenols

**Figure 1:**
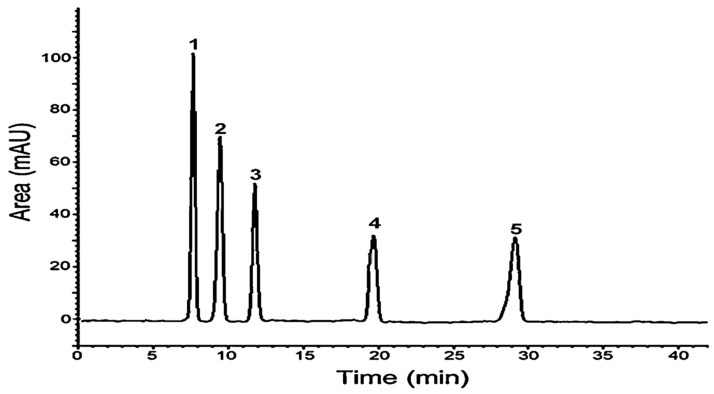
HPLC analysis of loquat juice polyphenols: 1: caffeic acid, 2: neochlorogenic acid, 3: chlorogenic acid, 4: quercetin, and 5: rutin.

The chromatogram shown in Figure 1 offers specific insights into the phenolic composition of *Mkerkeb* loquat juice. It reveals distinct peaks representing five identifiable phenolic compounds: peak 1 (caffeic acid 34%), peak 2 (neochlorogenic acid 27%), peak 3 (chlorogenic acid 17%), peak 4 (quercetin 10%), and peak 5 (rutin 10%).

### 3.3. Effect of Mkarkeb Juice on Plasma Lipid Profile in HFFD-Fed Mice

The impact of *Mkarkeb* loquat juice at 3.5 mL kg^−1^ and 7 mL kg^−1^ on plasma lipid parameters was evaluated across all mice groups at intervals of 2, 4, and 6 weeks (Table 2). In the hyperlipidemic control (HC) group, there was a notable increase in total cholesterol (TC) by 80% (*p* < 0.001), triglycerides (TG) by 36% (*p* < 0.001), low-density lipoprotein cholesterol (LDL-C) by 191% (*p* < 0.001), atherogenic index (AI) by 21% (*p* < 0.001), and LDL-C/HDL-C ratio by 582% (*p* < 0.001) observed as early as the second week of the study. These levels continued to rise significantly, reaching increases of 201% (*p* < 0.001), 146% (*p* < 0.001), 1037% (*p* < 0.001), 311% (*p* < 0.001), and 1185% (*p* < 0.001), respectively, by the end of six weeks of treatment. These results indicate the substantial effect of the high-fat, high-fructose diet (HFFD) on elevating plasma lipid levels. The decrease in high-density lipoprotein cholesterol (HDL-C) began subtly at week 2 and became statistically significant at week 4 with a 9% (*p* < 0.05).

Concerning the loquat-treated group, we observed that the juice at a 3.5 mL kg^−1^ dose on plasma lipids was insignificant throughout the treatment period. However, when the loquat juice dosage was increased to 7 mL kg^−1^, a substantial and pronounced effect on the plasma lipid profile was observed as early as 2 weeks into the treatment, with the impact being time-dependent. After 2 weeks, there were notable reductions in TC by 4.25% (*p* < 0.05), TG by 5.97% (*p* < 0.01), and LDL-C by 11.24% (*p* < 0.05) compared to the HC group. AI showed a significant decrease of 10% (*p* < 0.001), and the LDL-C/HDL-C ratio was reduced by 16% (*p* < 0.001). These positive changes persisted and even improved by week 4, with TC, TG, LDL-C, AI, and LDL-C/HDL-C ratios showing respective decreases of 7% (*p* < 0.001), 16% (*p* < 0.001), 12% (*p* < 0.05), 28% (*p* < 0.001), and 29% (*p* < 0.001). HDL-C levels rose by 9% (*p* < 0.001). By the end of the 6 weeks, the group receiving 7 mL kg^−1^ of loquat juice exhibited even more significant reductions: TC and TG levels decreased by 20% (*p* < 0.001) and 21% (*p* < 0.001), respectively, and LDL-C, AI, and LDL-C/HDL-C ratio were reduced by 22% (*p* < 0.001), 44% (*p* < 0.001), and 46% (*p* < 0.001) compared to the HC group. Furthermore, HDL-C levels saw a substantial increase of 45% (*p* < 0.001) compared to the HC group at 6 weeks.

For comparison, the standard hypolipidemic drug, fenofibrate, administered at a dose of 4 mg/kg, led to statistically significant reductions in plasma TC (14%, *p* < 0.01), TG (18%, *p* < 0.01), LDL-C (13%, *p* < 0.01), AI (30%, *p* < 0.001), and LDL-C/HDL-C ratio (35%, *p* < 0.001) after 4 weeks, along with a 33% (*p* < 0.001) increase in HDL-C levels.

As the study progressed to 6 weeks, fenofibrate continued to show impressive effects, with TC levels decreasing by 34% (*p* < 0.001) and TG levels by 37% (*p* < 0.001). LDL-C, AI, and LDL-C/HDL-C ratios experienced further reductions of 56% (*p* < 0.001), 54% (*p* < 0.001), and 68% (*p* < 0.001), respectively. HDL-C levels maintained their upward trend, increasing by 34% (*p* < 0.01). Notably, at a dose of 7 mL kg^−1^, the effect of the loquat juice on the lipid profile was comparable to that of fenofibrate.

It is worth mentioning that loquat juice administered to mice on a standard diet did not result in significant changes in lipid parameters compared to the NG group, emphasizing the specific impact of loquat juice on lipid metabolism disorders induced by the HFFD.

### 3.4. Effect of Mkarkeb Loquat Juice on the Plasma Glucose Level

The data presented in Table 2 show a significant increase in plasma glucose levels in the hyperlipidemic group, with a 57% rise (*p* < 0.001) compared to the normolipidemic group. Conversely, administering *Mkarkeb* loquat fruit juice to normolipidemic mice did not significantly change the glucose levels compared to the normolipidemic group (*p* > 0.05). However, in hyperlipidemic mice, loquat juice supplementation led to a dose-dependent decrease in plasma glucose. The JTG 3.5 mL kg^−1^ group showed no significant effect, while the JTG 7 mL kg^−1^ group experienced a substantial reduction of 12% (*p* < 0.01). Fenofibrate resulted in a more substantial reduction in plasma glucose levels, with a decrease of 33% (*p* < 0.01).

### 3.5. Effect of Mkarkeb Loquat Juice on Liver and Adipose Tissue Lipid in HFFD-Induced Hyperlipidemic Mice

Table 3 illustrates the impact of loquat juice on liver and adipose tissue lipid levels in mice subjected to an HFFD for 6 weeks. In the group of mice that received a standard diet and were administered loquat juice at a dosage of 7 mL kg^−1^, there was no significant change in liver and adipose tissue lipids compared to the normolipidemic group. However, the HFFD led to a significant increase in total cholesterol (TC) by 101% (*p* < 0.001) and triglycerides (TG) by 398% (*p* < 0.001) in the liver tissue of hyperlipidemic mice, compared to normolipidemic controls. Interestingly, loquat juice at 3.5 mL kg^−1^ alongside the HFFD showed no significant reduction in liver or adipose tissue lipids. However, at a higher dose of 7 mL kg^−1^, loquat juice demonstrated greater efficacy, leading to a significant decrease in hepatic TC by 41% (*p* < 0.001) and TG by 37% (*p* < 0.001). Additionally, it resulted in a reduction in adipose tissue TC by 41% (*p* < 0.001) and TG by 29% (*p* < 0.001). Similarly, the administration of fenofibrate significantly reduced hepatic TC by 40% (*p* < 0.001) and TG by 26% (*p* < 0.01), along with a significant decrease in adipose tissue TC by 38% (*p* < 0.001) and TG by 28% (*p* < 0.001).

### 3.6. Effect of Mkarkeb Loquat Juice on Biliary Cholesterol Excretion

**Figure 2:**
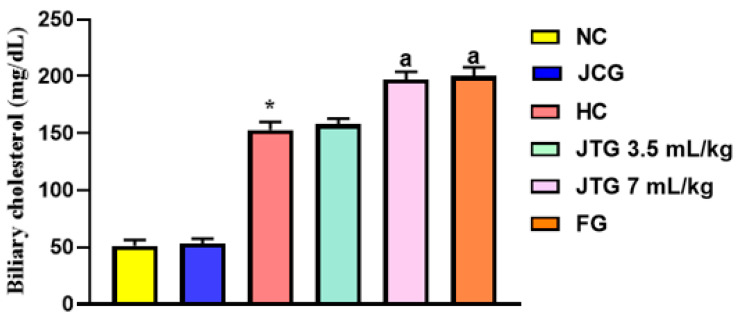
Effect of *Mkarkeb* loquat juice on biliary cholesterol in HFFD-induced hyperlipidemic mice. NC, normolipidemic control; HC, hyperlipidemic control; JCG, *Mkarkeb* loquat juice control group; JTG, *Mkarkeb* loquat juice-treated groups; FG, fenofibrate group. Each value is a mean ± SEM (n = 8). * *p* < 0.001 against NC. ^a^ *p* < 0.001 against HC.

Figure 2 illustrates the variations in biliary cholesterol levels across different treatment groups. Mice on an HFFD for six weeks showed a significant increase in bile cholesterol excretion, rising by 198% (*p* < 0.001) compared to the control group. However, administration of loquat juice at a dose of 7 mL kg^−1^ further increased biliary cholesterol excretion, with rises of 25% (*p* < 0.001) compared to the hyperlipidemic group. In contrast, the 3.5 mL kg^−1^ dose did not result in a significant increase compared to the hyperlipidemic group. Fenofibrate also produced a similar effect, increasing biliary cholesterol excretion by 32% (*p* < 0.001).

Loquat juice administered to normolipidemic mice maintained biliary cholesterol levels identical to those observed in the normolipidemic control group (*p* > 0.05).

### 3.7. Effect of Mkarkeb Loquat Juice on Fecal Cholesterol and Triglyceride Excretion

**Figure 3:**
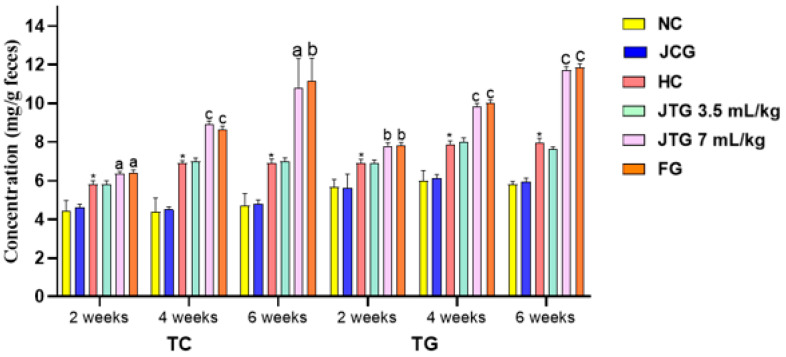
Effect of *Mkarkeb* loquat juice on excretion of fecal cholesterol and triglycerides. NC, normolipidemic control; HC, hyperlipidemic control; JCG, *Mkarkeb* loquat juice control group; JTG, *Mkarkeb* loquat juice-treated groups; FG, fenofibrate group. The values are means ± SEM (n = 8). * *p* < 0.001 against NC. ^a^ *p* < 0.05, ^b^ *p* < 0.01 and ^c^ *p* < 0.001 against HC.

Figure 3 displays the impact of loquat juice (3.5 mL kg^−1^ and 7 mL kg^−1^) on fecal cholesterol and triglyceride excretion. Mice on an HFFD exhibited a 31% (*p* < 0.001) increase in total cholesterol (TC) and a 22% (*p* < 0.001) increase in triglycerides (TG) in their feces after two weeks. These levels progressively increased throughout the 6-week treatment period compared to mice on a standard diet (NC).

Administering loquat juice at 7 mL kg^−1^ resulted in a notable rise in fecal TC and TG levels after two weeks, with increases of 14% (*p* < 0.05) and 11% (*p* < 0.01), respectively. The exact dosage led to a significant increase in fecal TC by 26% (*p* < 0.001) after four weeks and 46% (*p* < 0.001) after six weeks. TG excretion also significantly rose by 20% (*p* < 0.001) after four weeks, with a further increase to 48% (*p* < 0.001) after six weeks. Conversely, loquat juice at 3.5 mL kg^−1^ did not significantly affect TC and TG excretion alongside the period treatment. Fenofibrate enhanced TC and TG fecal excretion, showing comparable results to loquat juice. Specifically, it increased TC excretion by 25% (*p* < 0.001) and TG by 26% (*p* < 0.001) after four weeks. This effect became more pronounced after six weeks, with TC excretion rising by 61% (*p* < 0.01) and TG by 48% (*p* < 0.001). In contrast, the 3.5 mL kg^−1^ dose of loquat juice had no significant effect on fecal cholesterol and triglyceride levels.

### 3.8. Effect of Mkarkeb Loquat Juice on Food Intake and Body Weight

None of the experimental animals experienced mortality or exhibited any behavioral abnormalities throughout the study. Additionally, there were no significant differences in food intake among the various treatment groups, suggesting that neither loquat juice nor fenofibrate affected mice’s appetite on an HFFD, as detailed in Table 4. Compared to the normal group, mice treated with loquat juice showed no significant change in body weight, maintaining a weight level similar to that of healthy mice. In contrast, the hyperlipidemic group exhibited a gradual increase in body weight, with an 8% increase (*p* < 0.001) after 2 weeks, 30% increase (*p* < 0.001) after 4 weeks, and 33% increase (*p* < 0.001) after 6 weeks compared to the normal group. However, mice treated with loquat juice at a higher dose of 7 mL kg^−1^ experienced a reduction in body weight, with a 15% decrease (*p* < 0.001) after 4 weeks, and this reduction persisted after 6 weeks of treatment (*p* < 0.001), although no significant change was observed after the first 2 weeks. Similarly, the group treated with fenofibrate showed a 17% reduction (*p* < 0.001) in body weight after 4 weeks and an 18% reduction (*p* < 0.001) after 6 weeks compared to the hyperlipidemic group, as shown in Table 4. These findings demonstrate that the higher dose of loquat juice, like fenofibrate, effectively counteracted the weight gain typically associated with HFFD, contributing to a healthier body weight.

### 3.9. Effect of Mkarkeb Loquat Juice on Hepatic Injury Biomarkers in Mice

This study examined the impact of hyperlipidemia on liver function, a crucial organ involved in many vital physiological processes. Table 5 shows that mice fed an HFFD exhibited significant increases in several hepatic injury biomarkers: AST levels rose by 78% (*p* < 0.001), ALT by 127% (*p* < 0.001), ALP by 67% (*p* < 0.001), TB by 86% (*p* < 0.05), and LDH by 121% (*p* < 0.001) compared to the normal control group. Administering loquat juice at a dose of 3.5 mL kg^−1^ resulted in a slight, non-significant decrease in these biomarkers. However, a higher dose of 7 mL kg^−1^ effectively mitigated these elevations, reducing plasma AST levels by 10% (*p* < 0.05), ALT by 21% (*p* < 0.001), ALP by 22% (*p* < 0.001), TB by 41% (*p* < 0.001), and LDH by 9% (*p* < 0.001). Fenofibrate also showed significant reductions in these biomarkers: AST levels decreased by 14% (*p* < 0.01), ALT by 11% (*p* < 0.001), ALP by 30% (*p* < 0.001), TB by 40% (*p* < 0.05), and LDH by 35% (*p* < 0.001). Notably, the protective effects of *Mkarkeb* loquat juice at a dose of 7 mL kg^−1^ were comparable to those of fenofibrate, highlighting its potential efficacy in preventing hepatic injury associated with hyperlipidemia.

### 3.10. Effect of Mkarkeb Loquat Juice on Liver Lipid Peroxidation

**Figure 4:**
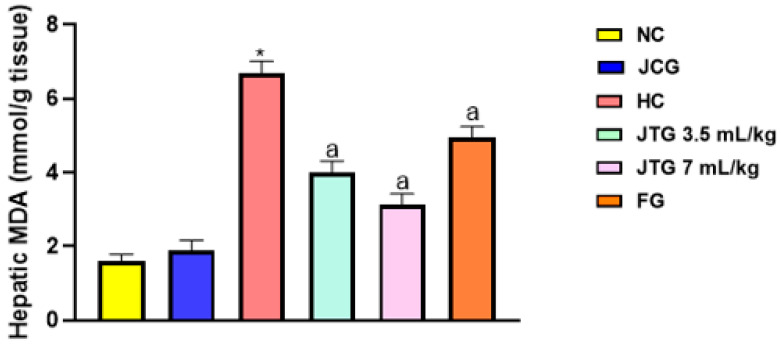
Effect of *Mkarkeb* loquat juice on liver lipid peroxidation in HFFD-fed mice. NC, normolipidemic control; HC, hyperlipidemic control; JCG, *Mkarkeb* loquat juice control group; JTG, *Mkarkeb* loquat juice-treated groups; FG, fenofibrate group; HFFD, high-fat–high-fructose diet. Data are mean± SEM (n = 8). * *p* < 0.001 against NC. ^a^ *p* < 0.001 against HC.

Malondialdehyde (MDA) formation, an indicator of lipid peroxidation resulting from the breakdown of polyunsaturated fatty acids, was utilized to assess oxidative stress in the livers of mice fed an HFFD. As shown in Figure 4, hyperlipidemic mice exhibited a dramatic increase in liver MDA levels, rising by over 318% (*p* < 0.001). A dose of 3.5 mL kg^−1^ of *Mkarkeb* loquat juice led to a significant reduction in MDA levels by 27% (*p* < 0.001), while a higher dose of 7 mL kg^−1^ resulted in a more substantial decrease of 39% (*p* < 0.001). In comparison, fenofibrate treatment resulted in a 26% reduction in lipid peroxidation (*p* < 0.01), although its effectiveness was still slightly lower than that of the higher dose of loquat juice.

On the other hand, when *Mkarkeb* loquat juice was administered to mice on a standard diet, it did not significantly affect liver lipid peroxidation compared to normolipidemic controls.

### 3.11. Effect of Mkarkeb Loquat Juice on Liver Antioxidant Enzymes Activities

**Figure 5:**
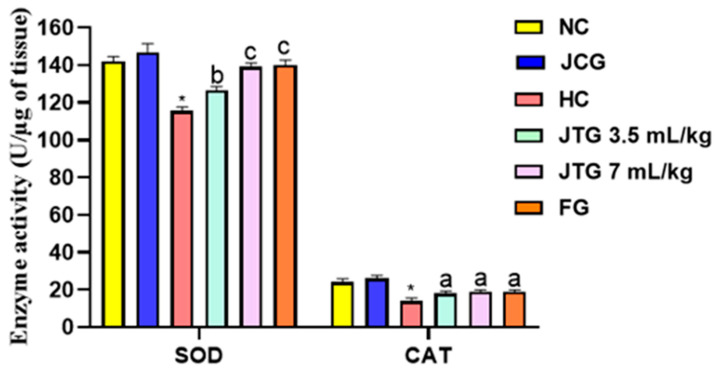
Effect of *Mkarkeb* loquat juice on superoxide dismutase (SOD) and catalase (CAT) activities in mice. NC, normolipidemic control; HC, hyperlipidemic control; JCG, *Mkarkeb* loquat juice control group; JTG, *Mkarkeb* loquat juice-treated groups; FG, fenofibrate group; HFFD, high-fat–high-fructose diet. Data are mean± SEM (n = 8). * *p* < 0.001 against NC. ^a^ *p* < 0.01; ^b^ *p* < 0.01 and ^c^ *p* < 0.001 against HC.

Based on Figure 5, HFFD feeding led to a significant fall in the liver SOD and catalase (CAT) activities in the hyperlipidemic group compared to the normolipidemic one, with reductions of 18% (*p* < 0.001) and 42% (*p* < 0.001), respectively. However, in treated groups, there was a significant rise in enzyme activities in a dose-dependent manner. *Mkarkeb* loquat juice, in particular, restored SOD and CAT activities by 12% (*p* < 0.01) and 22% (*p* < 0.05), respectively, at a dose of 3.5 mL kg^−1^, and by 25% (*p* < 0.001) and 36% (*p* < 0.05), respectively, at a dose of 7 mL kg^−1^. Furthermore, fenofibrate treatment increased SOD and CAT activity by approximately 21% (*p* < 0.001) and 36% (*p* < 0.05), respectively.

### 3.12. Effect of Mkarkeb Loquat Juice on Liver Morphology and Histology and Abdominal Adipose Tissue Accumulation

**Figure 6:**
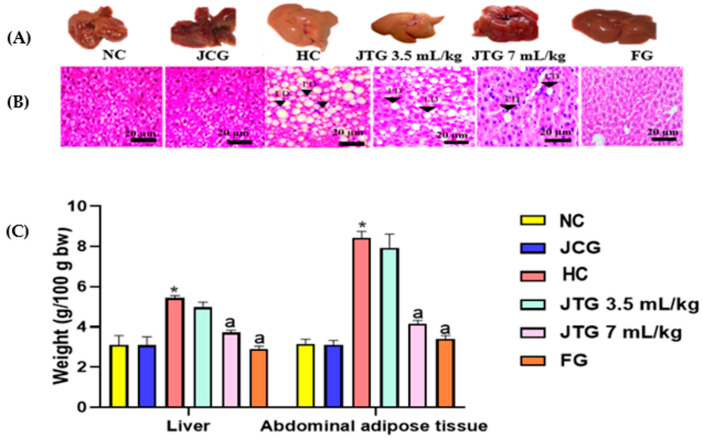
Effect of *Mkarkeb* loquat juice on liver and abdominal adipose tissue in HFFD-fed mice. (**A**), macroscopic aspect; (**B**), microscopic aspect; (**C**), liver and abdominal adipose tissue weight. NC, normolipidemic control; HC, hyperlipidemic control; JCG, *Mkarkeb* loquat juice control group; JTG, *Mkarkeb* loquat juice-treated groups; FG, fenofibrate group; HFFD, high-fat–high-fructose diet. Data are mean ± SEM (n = 8). * *p* < 0.001 against NC. ^a^ *p* < 0.001 against HC.

In Figure 6A, we visually compared the liver morphologies among different groups. The livers of the healthy control group displayed a glossy, sleek appearance with a dark red color, indicative of their normalcy. In contrast, the livers of hyperlipidemic mice appeared enlarged, with a yellowish tint and an irregular surface, suggesting the presence of a fatty liver condition. Following treatment with both loquat juice and fenofibrate, livers exhibited a reddish-brown appearance with smooth surfaces, resembling the livers in the control group. To provide detailed insights into the cellular changes underlying macroscopic observations, we present histological cross-sections of liver tissues observed under an optical microscope in Figure 6B. As can be observed, the livers from normolipidemic control mice showcased a regular appearance with well-organized hepatocytes, clear cell borders, violet nuclei, and ample cytoplasm. The picture is strikingly different in hyperlipidemic mice; their hepatocytes exhibited round and excessively swollen characteristics, signifying hepatocyte hyperplasia compared to normolipidemic mice.

Additionally, an abundance of empty cytoplasmic vacuoles of varying sizes was observed within the hepatocytes, previously identified as granular degeneration or lipid droplets. This suggests that HFFD induces a substantial influx of lipids into hepatocytes, accumulating lipid vacuoles. These observations are consistent with our results regarding elevated TC and TG levels in hyperlipidemic mice livers. In contrast to the hyperlipidemic group, mice treated with *Mkarkeb* loquat juice and fenofibrate exhibit dose-dependent mitigation of these histopathological modifications (quantity and dimensions of vacuoles and granular degeneration). These conclusions find further support in the corresponding liver lipid data, indicating lower TC and TG levels in the livers of *Mkarkeb* loquat juice-treated mice relative to hyperlipidemic ones.

Additionally, as depicted in Figure 6C, the relative liver weight of HFFD-fed mice was significantly higher than that of NC by 70% (*p* < 0.001). However, concurrent treatment with *Mkarkeb* loquat juice led to a reduction of this parameter by 15% (*p* < 0.01) and 31% (*p* < 0.001) at doses of 3.5 mL kg^−1^ and 7 mL kg^−1^, respectively. Notably, Fenofibrate exhibited a comparable effect, reducing relative liver weight by 46% (*p* < 0.001).

Additionally, a notable three-fold elevation (*p* < 0.001) in the relative weight of abdominal adipose tissue was observed in hyperlipidemic mice (Figure 6C). In contrast, a significant decrease in adipose tissue relative mass was observed in the group that received *Mkarkeb* loquat juice at doses of 7 mL kg^−1^ (49%, *p* < 0.001).

It is important to note also that the fenofibrate’s effect was comparable to that of *Mkarkeb* loquat juice (58%, *p* < 0.001) concerning decreasing the mass of adipose tissues.

The group treated with *Mkarkeb* loquat juice alone displayed no noteworthy disparities in livers’ macroscopic or microscopic characteristics. Furthermore, there were no discernible differences in the relative weights of the hepatic and adipose tissues. This suggests that the administration of *Mkarkeb* loquat juice did not significantly change the appearance or weight of these organs compared to the normolipidemic group.

## 4. Discussion

Lipid metabolism is crucial for maintaining the body’s structural integrity and signaling processes and preserving energetic balance [23]. However, this balance can be disrupted by diets high in fat and sugar, leading to fat accumulation in the liver and adipose tissues. Such dietary habits contribute to hyperlipidemia, oxidative stress, steatosis, weight gain, and an increased risk of cardiovascular diseases [24].

In contrast, diets rich in fruits and vegetables, like the Mediterranean diet, have been shown to confer health benefits primarily due to the synergistic properties of micronutrients and antioxidants [25]. These components are pivotal in reducing lipid levels, scavenging free radicals, and preventing lipid peroxidation [26].

Previously, our research demonstrated that loquat peel extract significantly reduced plasma TC and TG levels in hyperlipidemic mice, lowering their concentrations in bile and fecal matter [19]. In this study, we further investigated the nutritional composition of *Mkarkeb* loquat juice, focusing on its potential impact on lipid disorders in mice.

At its ripe stage, the *Mkarkeb* variety of loquat fruit contains a total soluble sugar content of 9.71 ± 0.07 g 100 g^−1^. This is consistent with findings from other studies on loquats from Morocco and elsewhere [2,27]. The main sugars found in many loquat varieties are sucrose, glucose, and fructose [28,29]. In our studied variety, fructose was the main sugar, followed by glucose, while sucrose was not detected. The high fructose content likely contributes to the *Mkarkeb* variety’s characteristic sweetness, which is highly favored in local markets compared to other varieties.

The flavor of loquat is also influenced by the balance between sweetness and acidity [5,30]. Optimal flavor in loquat varieties is typically achieved when the fruit has a total soluble solids (TSS) content between 10 and 12 °Brix and a titratable acidity of around 1 g of acid per 100 mL juice [31]. For the local *Mkarkeb* variety, we measured a TSS content of 12.40 ± 0.25° Brix and a titratable acidity of 0.64 ± 0.03%, with a TSS/TA ratio of 19.37 ± 0.17. These metrics explain why the *Mkarkeb* variety is so prevalent in the Berkane region. Additionally, *Mkarkeb* loquat contains proteins, lipids, and minerals in quantities similar to those found in other loquat varieties reported in the literature [3].

Beyond its macronutrient profile, *Mkarkeb* loquat’s richness in minor elements like carotenoids, ascorbic acid, and polyphenols potentially positions it as a fruit of exceptional nutritional quality and a functional food. In modern diets, the functional qualities of fruits are increasingly important to consumers, especially given evidence supporting the role of balanced fruit and vegetable intake in disease prevention [13]. Indeed, numerous studies have demonstrated the protective effects of micronutrient-rich fruits against hyperlipidemia and cardiovascular diseases [32,33].

In the present study, we realized that feeding mice with HFFD caused an increase in TC and LDL-C levels and a decrease in HDL-C levels in treated mice compared to the normolipidemic group. This imbalance negatively impacts the atherogenic index (AI) and the LDL-C/HDL-C ratio. However, treatment with *Mkarkeb* loquat juice for 6 weeks effectively reduced TC, particularly lowering the atherogenic LDL-C fraction. This suggests that *Mkarkeb* loquat juice may enhance LDL-C uptake by the liver and peripheral tissues through LDL receptors, as previously proposed [20].

The juice also positively impacted cholesterol metabolism by elevating the anti-atherogenic HDL-C fraction, which is crucial for reverse cholesterol transport (RCT) from peripheral tissues to the liver, where cholesterol is excreted into bile. The juice’s effect on the RCT pathway was evidenced by significant changes in cholesterol levels within the liver and bile of treated mice. Notably, mice receiving the juice had lower hepatic cholesterol and higher biliary cholesterol levels than controls, indicating adequate peripheral cholesterol clearance through the RCT pathway. These findings align with and extend previous research on loquat’s benefits against lipid metabolism disorders [19,34].

Furthermore, the AI and LDL-C/HDL-C ratios are considered reliable predictors of atherosclerotic risk, underscoring the importance of maintaining these parameters at low levels [35]. Our study demonstrated that loquat juice significantly improved these atherogenic parameters in hyperlipidemic mice, underscoring its potential nutritional value in atherosclerosis prevention and other lipid metabolism disorders. This supports our earlier work on loquat peels’ effects on lipid metabolism [19].

Given that dyslipidemia often coexists with hypertriglyceridemia, which is closely associated with abdominal obesity, overweight, and liver diseases like steatosis, these conditions can lead to insulin resistance, hyperglycemia, diabetes mellitus, and cardiovascular disease (CVD) [36]. This study’s prolonged HFFD exposure led to an increase in triglyceridemia, glycemia, hepatic and adipose tissue TG content, body weight, and visceral adipose tissue mass. These findings corroborate numerous earlier studies utilizing this mouse model [37,38,39].

Conversely, concurrent administration of *Mkarkeb* loquat juice markedly reduced TG levels in plasma, liver, and adipose tissue while enhancing their excretion in feces. This suggests that the juice may promote the uptake and catabolism of TG-rich lipoproteins via lipoprotein lipase (LPL) activation, as previously hypothesized [40].

Moreover, mice treated with the juice experienced significant reductions in body weight, liver, and adipose mass, implying the juice’s potential to inhibit lipogenic enzymes in adipose tissue, such as fatty acid synthase (FAS) [41] and diacylglycerol acyltransferase 2 (DGAT2) [42], while simultaneously activating fatty acid oxidation enzymes like carnitine palmitoyltransferase 1 (CPT1) [43].

To deepen our analysis, comparing with other fruits from the Rosaceae family, such as apples and peaches, could provide insight into loquat’s unique hypolipidemic and hepatoprotective properties. In fact, Bobadoye et al. [44] demonstrated that the administration of African Star Apple (*Chrysophyllum albidum*) juice at doses of 3 mL, 6 mL, and 9 mL over 28 days effectively decreased TC, TG, LDL-C, VLDL-C, and AI levels while reducing liver lipid accumulation and associated damage in hyperlipidemic rats. Similarly, research by Noratto et al. [45] indicated that polyphenol-rich peach and plum juice positively impacted the plasma lipid profile by decreasing cholesterol, triglycerides, and LDL oxidation levels. Hyperlipidemia often leads to excessive lipid deposition in the liver, causing cellular damage and inflammatory injuries, a phenomenon known as “lipotoxicity” [46]. In our study, chronic hyperlipidemia induced by HFFD altered liver color, morphology, and histology, indicating steatosis and lipotoxicity. This was corroborated by increased hepatic lipid content and elevated plasma markers of liver injury in hyperlipidemic mice (ALT, AST, ALP, LDH, and TB). However, after treatment with loquat fruit juice, we concluded that it provided significant protection against liver damage induced by lipotoxicity, as shown by the correction of plasma biochemical markers and the integrity of hepatic histological structures. This finding confirms our previous result on the protective effect of loquat peels against lipotoxicity [19]. It aligns with those reported by Shahat et al. [47], demonstrating the hepatoprotective effect of loquat leaves.

The observed protective effect of *Mkarkeb* loquat juice against chronic hyperlipidemia-induced hepatic tissue damage could be attributed to its potent hypolipidemic activity. The juice eliminates excess lipids in bile and fecal matter, preventing their harmful accumulation in liver tissue. Without this removal, accumulated lipids in the liver can undergo peroxidation, producing toxic molecules such as oxidized LDL and lipid free radicals, which can damage cell membranes and cause tissue injury [48]. In this regard, we demonstrated that *Mkarkeb* loquat juice mitigates liver oxidative stress by reducing MDA content and activating antioxidant enzymes, such as SOD and catalase. This aligns with previous studies highlighting the antioxidant properties of loquat seeds, leaves, and peels [19,49,50].

The protective effect of *Mkarkeb* loquat juice is likely due to the presence of beneficial minor compounds, such as phenolics and carotenoids, known for their pharmacological activities [51]. Fruit juice is rich in phenolics, particularly phenolic acids and flavonoids, which have been identified as natural alternatives for treating and preventing against several ailments without any side effects [20,52]. Additionally, these compounds, along with carotenoids and ascorbic acid, play crucial roles in combating oxidative stress at the tissue level [13,53]. Polyphenol analysis revealed that the juice contains five major phenolic compounds: neochlorogenic acid, chlorogenic acid, quercetin, and rutin, with caffeic acid being the predominant compound. All these phenolic compounds are known for their various pharmacological properties [54,55]. Our results disagree with those reported by Xu et al. [56] and Ding et al. [57], who identified chlorogenic acid as the main phenolic acid in Chinese and Japanese loquat fruits, respectively. These compounds can restore lipid metabolism and prevent fat accumulation in tissues and subsequent oxidation, leading to cellular damage.

In fact, as previously reported, polyphenols from apple extract were shown to modulate cholesterol metabolism via downregulation of the expression of two significant liver genes, farnesoid x receptor (FXR) and cytochrome P450 family 7 subfamily A member 1 (Cyp7A1) involved in cholesterol homeostasis [58]. Furthermore, these phytochemicals could regulate the expression of cholesteryl ester transfer protein (CETP) and proprotein convertase subtilisin/kexin type 9 (PCSK-9) directly implicated in cholesterol metabolism [59]. In the same vein, apple polyphenols were demonstrated to be able to regulate the expression of LDL-receptors (LDL-R) and sterol 12 α hydroxylase (CYP8B1), the mechanism by which these compounds can restore lipid metabolism disturbed by a high cholesterol diet [60]. In addition, it was demonstrated that black raspberry fruit regulates lipid metabolism via gene expression of 3-hydrozy-3-methylglutaryl-CoA (HMG-CoA) reductase involved in cholesterol biosynthesis, CYP7A1 responsible for bile acid synthesis, and LDL-R implicated in the uptake of cholesterol by the liver and peripheral tissues [61].

The results from our study on *Mkarkeb* loquat juice indicate potential health benefits linked to lipid metabolism. To relate these findings to human health, existing research highlights the effects of similar metabolites. For example, chlorogenic acid has been shown to lower total cholesterol and LDL levels in humans [62]. Similarly, quercetin is linked to improved lipid profiles in overweight individuals [63]. Thus, the bioactive compounds in *Mkarkeb* loquat juice may provide cardiovascular benefits and protection against conditions like hyperlipidemia and non-alcoholic fatty liver disease, warranting further clinical investigation.

## 5. Conclusions

The *Mkarkeb* variety of loquat fruit grown in eastern Morocco stands out as a nutritionally rich and flavorful option, making it a valuable addition to a balanced diet. Its optimal TSS/TA ratio, low-calorie profile, and abundance of essential minerals, carotenoids, ascorbate, and polyphenols highlight its beneficial effects on hyperlipidemia, hepatic steatosis, and oxidative stress. These findings indicate that incorporating *Mkarkeb* loquat fruit into the diet may be crucial in preventing hyperlipidemia and related cardiovascular diseases.

## Figures and Tables

**Table 1:** The physical characteristics, flavor profile, and nutritional composition of *Mkerkeb* loquat juice.

	Parameters	Mature
	Weight (g)	68.73 ± 2.19
	Length (mm)	41.27 ± 1.30
	Width (mm)	56.09 ± 2.44
	Fruit thickness (mm)	24.08 ± 1.94
	Fruit shape index	0.73 ± 0.13
	pH	3.71 ± 0.22
	Titrable acidity (TA) %	0.64 ± 0.03
	Total soluble solids (TSS) (°Brix)	12.40 ± 0.25
	TSS/TA	19.37 ± 0.17
	Total sugars (g 100 g^−1^)	9.71 ± 0.07
Sugars	Fructose (g 100 g^−1^)	4.72 ± 0.28
	Glucose (g 100 g^−1^)	2.44 ± 0.34
	Sucrose (g 100 g^−1^)	Not found
	Sweetness index (SI)	16.15 ± 0.31
	Fat (g 100 g^−1^)	0.052 ± 0.0029
	Protein (g 100 g^−1^)	0.38 ± 0.04
	Crude fiber (g 100 g^−1^)	0.11 ± 0.06
Organic acids	Malic acid (mg 100 g^−1^)	537.08 ± 11.04
	Tartaric acid (mg 100 g^−1^)	48.02 ± 3.14
	Succinic acid (mg 100 g^−1^)	16.10 ± 1.84
	Oxalic acid (mg 100 g^−1^)	9.10 ± 1.06
	Vitamin C (mg 100 g^−1^)	7.10 ± 0.08
	Carotenoids (µg g^−1^)	58.01 ± 4.05
	Ash (mg 100 g^−1^)	0.43 ± 0.02
	Potassium (mg 100 g^−1^)	259.01 ± 2.01
	Sodium (mg 100 g^−1^)	59.07 ± 1.40
	Phosphorus (mg 100 g^−1^)	23.10 ± 1.50
	Calcium (mg 100 g^−1^)	19.10 ± 1.01
	Magnesium (mg 100 g^−1^)	18.29 ± 0.99
	Iron (mg 100 g^−1^)	2.91 ± 0.10
	Energy Value (Kcal 100 g^−1^)	40.82 ± 0.25
	Total polyphenols	138.08 ± 0.71 ^a^
	Flavonoids	65.18 ± 4.53 ^b^

^a^ mg of catechin per 100 mL, ^b^ mg of rutin per 100 mL, and the results are presented as mean ± SEM (n = 3).

**Table 2:** *Mkarkeb* loquat juice’s effects on glucose and plasma lipids over time in mice.

Treatments	Parameters	0 Weeks	2 Weeks	4 Weeks	6 Weeks
NC	Total cholesterol (mg dL^−1^)	82.10 ± 1.04	82.46 ± 1.50	83.06 ± 1.12	84.04 ± 2.09
	Triglycerides (mg dL^−1^)	45.12 ± 1.07	46.18 ± 1.05	49.27 ± 1.08	50.01 ± 1.01
	LDL-C (mg dL^−1^)	8.57 ± 1.28	8.88 ± 2.51	8.21 ± 2.06	8.10 ± 1.43
	HDL-C (mg dL^−1^)	18.17 ± 0.99	18.10 ± 0.45	19.08 ± 0.49	19.09 ± 0.52
	AI	3.51 ± 0.79	3.55 ± 0.60	3.53 ± 0.14	3.40 ± 0.09
	LDL-C/HDL-C ratio	0.47 ± 0.15	0.49 ± 0.17	0.43 ± 0.05	0.42 ± 0.05
	Glucose (mg dL^−1^)	96.55 ± 3.19	98.18 ± 6.50	99.50 ± 3.09	100.1 ± 1.12
JCG	Total cholesterol (mg dL^−1^)	82.7 ± 1.21	79.18 ± 2.44	80.20 ± 3.87	83.10 ± 4.77
	Triglycerides (mg dL^−1^)	49.1 ± 3.17	49.10 ± 1.70	50.07 ± 1.11	51.90 ± 1.21
	LDL-C (mg dL^−1^)	8.37 ± 1.24	8.78 ± 1.31	7.95 ± 2.61	7.99 ± 2.44
	HDL-C (mg dL^−1^)	19.05 ± 0.36	19.08 ± 0.77	20.40 ± 1.88	20.10 ± 3.40
	AI	2.98 ± 0.71	3.14 ± 0.33	2.93 ± 0.73	3.14 ± 0.61
	LDL-C/HDL-C ratio	0.40 ± 0.10	0.46 ± 0.29	0.38 ± 0.31	0.39 ± 0.81
	Glucose (mg dL^−1^)	95.34 ± 5.10	99.03 ± 3.93	100.09 ± 4.88	101.1 ± 12
HC	Total cholesterol (mg dL^−1^)	81.10 ± 2.09	145.19 ± 2.17 ***	188.3 ± 1.12 **	251.70 ± 3.84 **
	Triglycerides (mg dL^−1^)	51.29 ± 1.32	68.08 ± 1.14 ***	99.8 ± 1.70 **	129.18 ± 1.61 **
	LDL-C (mg dL^−1^)	8.09 ± 3.17	58.70 ± 1.70 ***	57.18 ± 2.11 **	92.81 ± 1.51 **
	HDL-C (mg dL^−1^)	20.02 ± 1.90	19.12 ± 0.82	18.18 ± 0.79 *	18.03 ± 0.19 *
	AI	3.19 ± 0.39	6.59 ± 0.17 ***	9.35 ± 0.28 **	12.96 ± 1.09 **
	LDL-C/HDL-C ratio	0.30 ± 0.17	3.07 ± 0.09 ***	3.14 ± 0.05 **	5.14 ± 0.08 **
	Glucose (mg dL^−1^)	100.09 ± 4.09	103.77 ± 5.19	106 ± 3.62	171.09 ± 6.10 ***
JTG 3.5 mL kg^−1^	Total cholesterol (mg dL^−1^)	84.12 ± 3.11	143.99 ± 1.41	186.22 ± 3.10	249.11 ± 3.81
	Triglycerides (mg dL^−1^)	52.22 ± 1.19	67.26± 1.54	102.10 ± 2.71	127.12 ± 2.03
	LDL-C (mg dL^−1^)	8.16 ± 1.05	58.91 ± 0.63	59.11 ± 1.11	89.99 ± 2.53
	HDL-C (mg dL^−1^)	20.10 ± 1.32	20.09 ± 1.21	20.03 ± 2.07	20.98 ± 2.94
	AI	3.18 ± 0.11	6.16 ± 1.95	8.29 ± 1.12	10.87 ± 1.33
	LDL-C/HDL-C ratio	0.40 ± 0.19	2.94 ± 0.08	2.95 ± 0.19	4.28 ± 0.53
	Glucose (mg dL^−1^)	101.18 ± 4.11	99.81 ± 7.44	101.01 ± 7.90	169.01 ± 3.01
JTG 7 mL kg^−1^	Total cholesterol (mg dL^−1^)	84.77 ± 1.31	139.01 ± 1.15 ^a^	176.12 ± 1.46 ^c^	199.01 ± 2.10 ^c^
	Triglycerides (mg dL^−1^)	52.92 ± 1.87	64.01 ± 0.23 ^b^	87.10 ± 1.04 ^c^	101.72 ± 2.01 ^c^
	LDL-C (mg dL^−1^)	8.29 ± 1.84	52.10 ± 0.05 ^a^	52.08 ± 0.46 ^a^	72.41 ± 1.06 ^c^
	HDL-C (mg dL^−1^)	20.17 ± 1.39	20.21 ± 0.21	23.04 ± 0.22 ^c^	26.17 ± 1.18 ^c^
	AI	3.20 ± 0.18	5.87 ± 0.10 ^c^	6.64 ± 0.06 ^c^	7.15 ± 0.04 ^c^
	LDL-C/HDL-C ratio	0.40 ± 0.04	2.57 ± 0.07 ^c^	2.26 ± 0.08 ^c^	2.76 ± 0.09 ^c^
	Glucose (mg dL^−1^)	102.12 ± 4.10	101.81 ± 4.44	102.07 ± 6.06	149.1 ± 4.2 ^b^
FG	Total cholesterol (mg dL^−1^)	83.19 ± 2.66	140.01 ± 1.71	181.5 ± 1.37 ^b^	164.5 ± 1.51 ^c^
	Triglycerides (mg dL^−1^)	50.74 ± 2.23	64.1 ± 2.07	91.70 ± 1.90 ^b^	76.02 ± 1.11 ^c^
	LDL-C (mg dL^−1^)	8.19 ± 1.47	55.92 ± 1.60	49.60 ± 1.27 ^b^	39.19 ± 1.46 ^c^
	HDL-C (mg dL^−1^)	21.91 ± 1.71	21.23 ± 1.09	24.27 ± 1.71 ^c^	24.01 ± 1.64 ^b^
	AI	2.79 ± 0.43	5.59 ± 1.45	6.47 ± 0.09 ^c^	5.85 ± 0.07 ^c^
	LDL-C/HDL-C ratio	0.37 ± 0.11	2.63 ± 0.30	2.04 ± 0.28 ^c^	1.63 ± 0.05 ^c^
	Glucose (mg dL^−1^)	104.16 ± 4.19	107.16 ± 4.08	108.15 ± 5.01	121.06 ± 11 ^b^

NC, normolipidemic group; HC, hyperlipidemic control; JCG, *Mkarkeb* loquat juice control group; JTG, *Mkarkeb* loquat juice-treated groups; FG, fenofibrate group. The results are expressed as mean ± SEM (n = 8). * *p* < 0.05, ** *p* < 0.01 and *** *p* < 0.001 vs. NC, ^a^ *p* < 0.05, ^b^ *p* < 0.01, ^c^ *p* < 0.001 against HC.

**Table 3:** Liver and adipose tissue lipid profile modulation by *Mkarkeb* loquat juice in HFFD-induced hyperlipidemic mice.

	Parameters (mg g^−1^)	NC	JCG	HC	JTG 3.5 mL kg^−1^	JTG 7 mL kg^−1^	FG
Liver	TC	10.26 ± 0.61	10.77 ± 0.76	20.64 ± 1.51 *	18.15 ± 2.71	12.10 ± 0.40 ^b^	12.27 ± 0.24 ^b^
	TG	4.56 ± 0.67	4.53 ± 0.98	22.71 ± 1.76 *	23.15 ± 0.75	14.17 ± 0.27 ^b^	16.76 ± 0.41 ^a^
Adipose tissue	TC	1.89 ± 0.12	1.91 ± 0.15	3.19 ± 0.17 *	2.41 ± 0.20	1.86 ± 0.21 ^b^	1.97 ± 0.10 ^b^
	TG	7.56 ± 0.68	7.77 ± 0.81	27.71 ± 0.29 *	25.14 ± 1.21	19.61 ± 1.21 ^b^	20.12 ± 0.54 ^b^

NC, normolipidemic control; HC, hyperlipidemic control; JCG, *Mkarkeb* loquat juice control group; JTG, *Mkarkeb* loquat juice-treated groups; FG, fenofibrate group; HFFD, high-fat–high-fructose diet. Data are mean ± SEM (n = 8). * *p* < 0.001 against NC. ^a^ *p* < 0.05 and ^b^ *p* < 0.01 against HC.

**Table 4:** Effect of *Mkarkeb* loquat juice on food intake and body weight in HFFD-induced hyperlipidemic mice.

	2 Weeks		4 Weeks		6 Weeks	
	BW (g)	FI (g/day)	BW (g)	FI (g/day)	BW (g)	FI (g/day)
NC	28.03 ± 0.12	2.19 ± 0.06	28.46 ± 0.51	2.05 ± 0.04	28.46 ± 0.51	2.02 ± 0.12
JCG	28.17 ± 0.23	2.27 ± 0.18	28.81 ± 0.37	2.12 ± 0.10	28.81 ± 0.37	2.20 ± 0.09
HC	29.95 ± 0.24 *	2.05 ± 0.32	35.41 ± 1.18 *	2.15 ± 0.19	35.41 ± 1.18 *	2.35 ± 0.08
JTG 3.5 mL kg^−1^	29.51 ± 0.97	2.11 ± 0.81	35.17 ± 1.27	2.05 ± 0.72	35.17 ± 1.27	2.02 ± 0.51
JTG 7 mL kg^−1^	29.01 ± 0.41	2.13 ± 0.42	30.11 ± 0.40 ^a^	2.03 ± 0.14	30.11 ± 0.40 ^a^	2.03 ± 0.22
FG	28.30 ± 1.21	2.16 ± 0.12	29.07 ± 1.09 ^a^	2.07 ± 0.21	29.07 ± 1.09 ^a^	2.22 ± 0.31

BW: Body weight (g/animal), FI: Food intake (g/animal/day), NC, normolipidemic control; HC, hyperlipidemic control; JCG, *Mkarkeb* loquat juice control group; JTG, *Mkarkeb* loquat juice-treated groups; FG, fenofibrate group; HFFD, high-fat–high-fructose diet. All values represent the mean ± SEM. * *p* < 0.001 against NC. ^a^ *p* < 0.001 against HC.

**Table 5:** Effect of *Mkarkeb* loquat juice on various biomarkers of hepatic injury in HFFD-induced hyperlipidemic mice.

	AST (U L^−1^)	ALT (U L^−1^)	ALP (U L^−1^)	TB (mg dL^−1^)	LDH (U L^−1^)
NC	53.79 ± 1.94	39.18 ± 2.73	59.71 ± 0.96	1.87 ± 0.74	61.74 ± 1.91
JCG	52.1 ± 2.14	38.21 ± 2.17	58.19 ± 1.91	1.53 ± 0.70	60.71 ± 1.41
HC	96.07 ± 3.19 **	89.14 ± 3.19 **	99.73 ± 1.71 **	3.49 ± 0.09 *	137.17 ± 2.40 **
JTG 3.5 mL kg^−1^	95.84 ± 2.08	86.77 ± 2.17	99.07 ± 2.09	2.51 ± 0.78	132.11 ± 2.09
JTG 7 mL kg^−1^	85.71 ± 3.01 ^a^	70.15 ± 2.44 ^c^	78.01 ± 1.79 ^c^	2.05 ± 0.14 ^c^	126.00 ± 1.01 ^c^
FG	82.42 ± 1.19 ^b^	73.25 ± 2.09 ^c^	86.19 ± 1.67 ^c^	2.22 ± 0.33 ^b^	111.89 ± 2.10 ^c^

AST, aspartate aminotransferase; ALT, alanine aminotransferase; ALP, alkaline phosphatase; TB, total bilirubin; LDH, lactate dehydrogenase; NC, normolipidemic control; HC, hyperlipidemic control; JCG, *Mkarkeb* loquat juice control group; JTG, *Mkarkeb* loquat juice-treated groups; FG, fenofibrate group; HFFD, high-fat–high-fructose diet. The values are means ± SEM (n = 8). * *p* < 0.05 and ** *p* < 0.001 against NC. ^a^ *p* < 0.05, ^b^ *p* < 0.01 and ^c^ *p* < 0.001 against HC.

## Data Availability

Data are contained within the article.
